# Model-Informed Speech Enhancement Using Virtual Room Acoustics and Acoustic Descriptor Optimization

**DOI:** 10.3390/s26123630

**Published:** 2026-06-06

**Authors:** Samuel Yaw Mensah, Tao Zhang, Xin Zhao, Nahid-Al Mahmud

**Affiliations:** 1 School of Electrical & Information Engineering, Tianjin University, 92 Weijin Road, Nankai District, Tianjin 300072, China; nahidalmahmud@tju.edu.cn; 2 Digital Signal Processing Laboratory, Tianjin University, 92 Weijin Road, Nankai District, Tianjin 300072, China; zhangtao@tju.edu.cn (T.Z.); zhaoxin_16@tju.edu.cn (X.Z.)

**Keywords:** speech enhancement, dereverberation, room acoustics, hybrid modeling, Helmholtz equation, DRR, C50, physics-informed signal processing

## Abstract

Reverberation and background noise remain persistent obstacles to achieving clear and intelligible speech in enclosed environments. Conventional data-driven or purely empirical dereverberation systems often perform well only under training conditions but lack robustness and physical interpretability when exposed to new acoustic spaces. To address these limitations, this paper proposes a physics-informed speech enhancement algorithm that integrates analytical room acoustics modeling with a descriptor-guided optimization framework. The method employs virtual field simulations based on the Helmholtz equation to estimate key acoustic descriptors, reverberation time (RT60), direct-to-reverberant ratio (DRR), and clarity index (C50), which are then used to adaptively control a model-informed dereverberation filter. This hybrid formulation bridges physical modeling and signal processing, allowing the algorithm to minimize late reverberation energy while maintaining spectral fidelity. Experimental results across multiple simulated and real-room conditions demonstrate measurable improvements over baseline methods, achieving average gains of +6.4 dB in SNR, +1.2 in PESQ, and +0.13 in STOI, along with reduced RT60 and enhanced clarity. The proposed approach offers both computational efficiency and interpretability, making it suitable for real-time deployment in teleconferencing, hearing-assistive, and smart audio applications.

## 1. Introduction

Speech enhancement in reverberant and noisy environments remains one of the most critical challenges in audio signal processing. When speech propagates within enclosed spaces, reflections from walls, ceilings, and other surfaces combine with ambient noise to degrade intelligibility and perceived quality [1]. Traditional signal-processing methods often struggle to handle these distortions, particularly when room geometry or material properties vary significantly.

Recent advances in deep neural networks (DNNs) and transformer-based architectures have produced impressive results in dereverberation and denoising tasks [2]. However, such data-driven approaches tend to generalize poorly to unseen acoustic environments because they rely heavily on the characteristics of the training data. Conversely, purely physics-based simulations provide accurate acoustic representations but lack adaptability and computational efficiency for real-time applications [3,4].

To overcome these limitations, this study proposes a model-informed speech enhancement framework that integrates physically meaningful acoustic descriptors into a real-time dereverberation algorithm. By combining wave-based room modeling with descriptor-guided optimization, the proposed approach enhances speech clarity while maintaining interpretability and low computational cost.

The main contributions of this paper are as follows:

- Derivation of a model-informed dereverberation filter H(f) using RT_60_ and DRR as physical priors.
- Development of a virtual field experiment framework for acoustic parameter calibration.
- Comparative evaluation against deep neural network (DNN) and weighted prediction error (WPE) baselines.
- Statistical validation of descriptor accuracy across diverse materials and room geometries.

**Figure 1:**
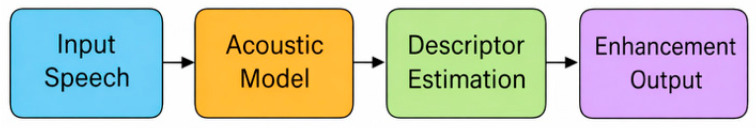
System overview block diagram.

Figure 1 presents the overall system architecture, showing the flow from input speech through the acoustic model and descriptor estimation to the enhancement filter that produces the final enhanced output.

Despite significant progress in both data-driven and physics-based approaches, a fundamental gap remains in the explicit integration of physically interpretable acoustic descriptors into real-time adaptive filtering frameworks. Existing methods either lack generalization across environments or fail to provide interpretable control parameters.

This work addresses this gap by introducing a descriptor-guided optimization framework that directly links measurable acoustic properties (RT60, DRR, C50) to adaptive filter design, enabling both robustness and physical interpretability in speech enhancement.

## 2. Related Work

Research on speech enhancement and dereverberation has evolved through several distinct approaches, each with its own advantages and limitations. These methods can be broadly categorized into simulation-based room acoustics modeling, statistical dereverberation, deep learning-based enhancement, and physics-informed hybrid frameworks.

### 2.1. Simulation-Based Room Acoustics

Classical room acoustics research focuses on physically modeling sound propagation within enclosed spaces using deterministic or numerical methods. The image source method models early reflections by mirroring sound sources across room boundaries, effectively capturing geometric reflections with low computational cost [5]. However, it fails to account for diffuse reverberation at higher frequencies. The finite-difference time-domain (FDTD) approach and ray-tracing methods improve accuracy by directly solving the acoustic wave equation, allowing more realistic modeling of material absorption and scattering [6]. Despite their physical interpretability, these methods are computationally expensive and impractical for real-time applications [7].

### 2.2. Statistical Dereverberation

Statistical models treat reverberation as a stochastic process superimposed on clean speech. The weighted prediction error (WPE) algorithm estimates and suppresses late reverberation by predicting and subtracting delayed signal components in the time–frequency domain [8]. Similarly, spectral subtraction and Kalman filtering approaches model noise and reverberation as additive random variables, using statistical priors to estimate the clean signal [9,10]. These methods perform well for stationary reverberation but degrade under dynamically changing room conditions, as they lack awareness of physical room characteristics such as geometry or boundary impedance [11].

### 2.3. Deep Learning-Based Enhancement

Modern data-driven methods have achieved strong dereverberation and noise suppression performance. Architectures such as DAE, U-Net, and transformer-based models have demonstrated strong performance in speech enhancement tasks by learning complex mappings from noisy to clean speech representations [12]. While these networks can outperform traditional methods in matched conditions, they suffer from poor generalization to unseen rooms or materials Their black-box nature also limits interpretability, and training large networks demands extensive labeled datasets covering diverse acoustic configurations [13].

### 2.4. Physics-Informed and Hybrid Frameworks

Recent efforts have sought to bridge physical modeling and data-driven learning by embedding physical constraints or acoustic descriptors into neural architectures. Physics-informed neural networks (PINNs) and hybrid acoustic models incorporate governing equations, such as the Helmholtz equation, into the learning process. These approaches improve generalization and interpretability but often stop short of explicitly integrating analytical acoustic descriptors, such as reverberation time (RT_60_), direct-to-reverberant ratio (DRR), and clarity index (C_50_), into adaptive signal-processing algorithms [14].

#### Research Gap

Despite progress in each domain, no existing method directly couples measurable acoustic descriptors (RT_60_, DRR, C_50_) with adaptive dereverberation filter design. The proposed work addresses this gap by formulating a model-informed enhancement algorithm that leverages these descriptors to guide real-time signal processing, achieving both physical interpretability and computational efficiency. A comparative summary of these different methodologies is presented in Table 1.

## 3. Theoretical Framework

### 3.1. Acoustic Modeling Fundamentals

The acoustic behavior of speech in enclosed spaces can be described by the linear wave equation derived from the conservation of mass and momentum under small-signal assumptions. For a homogeneous and isotropic medium, the sound pressure p(r,t) satisfies the following relation [15]:

∇^2^p(r,t) − (1/c^2^) * (∂^2^p(r,t)/∂t^2^) = 0 (1)

where c represents the speed of sound in air and r denotes spatial coordinates.

At the room boundaries, sound waves interact with surfaces whose impedance determines how much energy is absorbed or reflected. The boundary condition is expressed as

p = Zb * vn(2)

where Zb is the surface impedance and vn is the normal particle velocity. These equations collectively describe how the direct sound wave and its reflections form a reverberant field that influences speech clarity [16].

Acoustic descriptors such as reverberation time (RT60), direct-to-reverberant ratio (DRR), and clarity index (C50) are used to quantify these effects. RT60 measures the time it takes for the sound energy to decay by 60 dB after the source stops, indicating the persistence of reverberation [17]. DRR describes the energy balance between the direct and reflected components, serving as a proxy for spatial intelligibility [18]. C50 measures the ratio between early and late arriving energy, linking directly to perceived speech clarity.

**Figure 2:**
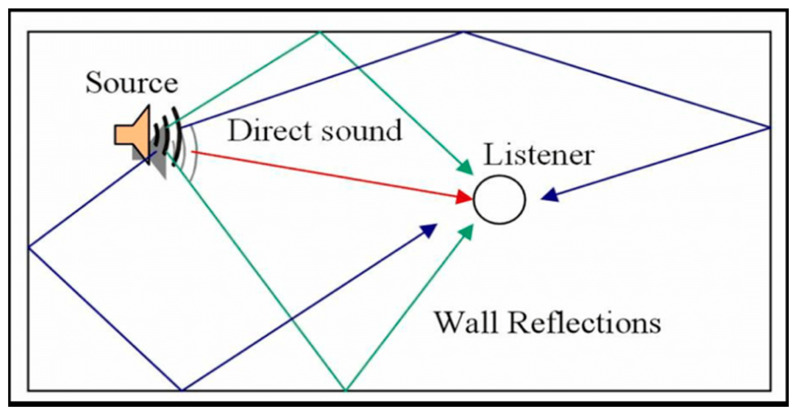
Diagram of sound propagation paths showing direct sound and reflected components from boundaries. The red arrow represents the direct sound path, while green and blue arrows indicate various reflected paths from room boundaries to the receiver.

Together, these parameters define the acoustic characteristics that determine intelligibility and guide optimization in speech enhancement algorithms. The interaction between these descriptors and the physical environment is illustrated in Figure 2, which depicts the primary propagation paths between a sound source and a listener.

### 3.2. Descriptor-Driven Speech Model

The received speech signal in a reverberant environment can be modeled as a convolution between the clean speech and the room’s impulse response, with additive noise:

x(t) = s(t) * h(t) + n(t)(3)

where s(t) represents the clean speech, h(t) encodes both early and late reflections, and n(t) denotes background noise.

The impulse response h(t) contains the essential temporal and spectral information of the acoustic environment. From h(t), the following energy-based descriptors can be derived:

RT60 = 0.161 * V/Aeq(4)

Aeq = Σ(αi * Si)

where V is the room volume, αi is the absorption coefficient, and Si is the surface area of the i-th boundary.

Although Equation (4) expresses RT60 in terms of room geometry and absorption properties, it is fundamentally linked to the temporal behavior of the room impulse response h(t). Specifically, RT60 characterizes the rate at which the energy of h(t) decays over time after the excitation signal has ceased.

In this context, the impulse response h(t) can be interpreted as an exponentially decaying function in its late reverberation region, where RT60 governs the decay slope. Therefore, Equation (4) provides a physical interpretation of how room properties influence the temporal structure of h(t), bridging analytical room acoustics and signal-domain modeling.

The direct-to-reverberant ratio (DRR) is expressed as:

DRR = 10 * log10(Ed/Er)(5)

where Ed and Er represent the energies of the direct and reverberant sound components, respectively.

The clarity index for speech, C50, quantifies the balance of early versus late arriving energy [19]:

(6) C50 = 10 ∗ log10(∫050 h2(t) dt/∫50^∞ h2(t) dt)

These descriptors can be linked to the modulation transfer function (MTF), which expresses how well amplitude modulations in speech are preserved through the acoustic channel [20]. The MTF can be defined as the ratio between the modulation depth at the output and the input:

MTF(fm) = m_out(fm)/m_in(fm)(7)

where fm denotes the modulation frequency. A high MTF value indicates better intelligibility, as more of the original modulation is preserved.

RT60 and DRR influence the temporal and energy distribution of the impulse response h(t), which in turn affects the preservation of amplitude modulations in speech signals. These modulations are quantified by the modulation transfer function (MTF), representing how well speech envelope fluctuations are transmitted through the acoustic channel.

A higher RT60 leads to increased temporal smearing, reducing modulation depth, while a lower DRR indicates stronger reverberant energy relative to direct sound, further degrading modulation clarity. Since the Speech Transmission Index (STI) is computed as an aggregate function of the MTF across frequency bands, changes in RT60 and DRR indirectly affect the STI by altering modulation fidelity [21].

A comprehensive summary of these acoustic descriptors, their governing equations, and their perceptual meanings is provided in Table 2.

In this framework, these descriptors act not only as analytical metrics, but also as adaptive parameters for guiding the enhancement algorithm introduced in the next section.

## 4. Proposed Model-Informed Speech Enhancement Algorithm

### 4.1. Problem Formulation

The primary objective of the proposed algorithm is to minimize the residual energy of late reverberation while preserving the spectral and temporal characteristics of the original clean speech. This optimization is guided by physically meaningful acoustic descriptors such as RT60, DRR, and C50 [22].

The problem can be formulated as:

(8) $$min\_\hat{s}(t)\;[||\;x(t)\;-\;\hat{s}(t)\;*\;h(t)\;||2\;+\;\lambda \;*\;\Phi (RT60,\;DRR,\;C50)]$$

where:

- x(t) is the observed reverberant and noisy signal,
- $\hat{s}(t)$ is the enhanced (dereverberated) speech estimate,
- h(t) represents the room impulse response,
- λ is a weighting parameter controlling the trade-off between distortion and dereverberation, and
- Φ(·) is a cost function that penalizes deviation of estimated descriptors from their target values.

The cost function Φ can be expressed as:

Φ(RT60, DRR, C50) = α_1_ * (RT60_est − RT60_ref)^2^ + α_2_ * (DRR_est − DRR_ref)^2^ + α_3_ * (C50_est − C50_ref)^2^ (9)

where α_1_, α_2_, and α_3_ are scaling coefficients that determine the relative importance of each descriptor term [23].

To establish a connection between the descriptor-based optimization objective in Equation (9) and the adaptive filter formulation, we relate the minimization of Φ to the suppression of late reverberation energy in the impulse response.

In reverberant environments, the late portion of the room impulse response can be approximated as an exponentially decaying process:

h_late(t) ≈ exp(−t/τ_r),

where τ_r is the reverberation decay constant, which is directly proportional to RT60. This implies that larger RT60 values correspond to slower decay and stronger late reverberation components.

Furthermore, the direct-to-reverberant ratio (DRR) governs the relative energy contribution between early (useful) and late (distorting) reflections. A lower DRR indicates stronger reverberation energy relative to the direct sound.

By expressing the residual reverberation energy in the frequency domain, the suppression strength required by the dereverberation filter can be modeled as a function of these descriptors. Therefore, minimizing Φ(RT60, DRR, C50) implicitly corresponds to adjusting the filter gain parameter β(f) such that late reverberation energy is attenuated while preserving early reflections.

However, due to the nonlinear dependence of Φ on the acoustic descriptors and the lack of a closed-form analytical solution, we approximate this relationship using a parametric mapping:

β(f)≈ f(RT60, DRR)

which is implemented as the regression model in Equation (11).

This approximation enables the descriptor-driven optimization objective to be translated into a practical adaptive filter design while maintaining physical interpretability and computational efficiency.

This formulation ensures that the algorithm adaptively suppresses late reflections while maintaining physically interpretable acoustic characteristics. The connection between this optimization objective and the filter parameterization is established through the descriptor-to-filter mapping described above [24].

### 4.2. Derivation of Adaptive Dereverberation Filter

To realize the above objective, an adaptive dereverberation filter is designed in the frequency domain. The filter operates as a model-informed correction term that dynamically adjusts to the acoustic conditions estimated from the room impulse response [25].

The discrete-frequency filter is defined as:

H(f) = 1/[1 + β(f) * e^(−j * 2π * f * τ(f))](10)

where:

- β(f) is the decay coefficient that governs the suppression strength of late reverberation,
- τ(f) is the average delay associated with the early reflection boundary, and
- j is the imaginary unit.

The filter formulation in Equation (10) was inspired by standard frequency-domain dereverberation models and was adapted in this study to incorporate descriptor-driven control. The exponential term models delayed reflections, while the gain parameter β(f) governs suppression strength.

The decay parameter β(f) is estimated using a calibration-based regression model derived from physically simulated acoustic conditions. It is important to note that this regression is not a supervised learning model trained on labeled datasets, but rather a parametric fitting procedure that maps acoustic descriptors (RT60, DRR) to filter parameters based on physically consistent simulations.

The calibration is performed using a subset of simulated room configurations, while evaluation is conducted on distinct acoustic scenarios and independently generated impulse responses, including those aligned with measured datasets (e.g., AIR database). This separation ensures that the reported performance reflects generalization across different acoustic conditions rather than the reuse of identical simulation data.

β(f) = a_1_ * exp(−b_1_ * DRR(f)) + a_2_ * RT60(f) (11)

where a_1_, a_2_, and b_1_ are regression coefficients determined through calibration using virtual field experiments [26].

The parametric form in Equation (11) is proposed in this study as a regression-based approximation linking acoustic descriptors to filter behavior. The coefficients are calibrated using virtual acoustic simulations, and their effectiveness is validated through the performance improvements reported in Section 6.

This parametric relationship ensures that the dereverberation gain adapts automatically to varying acoustic environments, improving performance in both diffuse and directional sound fields [27].

#### Descriptor-Guided Dereverberation (Pseudocode)

Input: Reverberant signal x(t), impulse response h(t)

Output: Enhanced signal $\hat{s}$(t)

1. Estimate acoustic descriptors:

Compute RT60_est, DRR_est, and C50_est from h(t).

2. Calculate β(f) and τ(f):

β(f) = a_1_ * exp(−b_1_ * DRR_est(f)) + a_2_ * RT60_est(f)

τ(f) = mean delay of early reflections (0–50 ms window).

3. Design dereverberation filter:

H(f) = 1/[1 + β(f) * e^(−j * 2π * f * τ(f))]

4. Apply filter in frequency domain:


$$\hat{s}(f)=H(f)\;*\;X(f)$$

$$\hat{s}(t)=IFFT(\hat{S}(f))$$

5. Update parameters iteratively:

β(f) ← β(f) + η * (Φ_target − Φ_current)

Repeat until convergence or maximum iterations reached.

where η is the learning rate controlling the adaptive update step.

In Step 4, the dereverberation filter H(f) is applied in the frequency domain by multiplying it with the Fourier transform of the input signal X(f). The enhanced signal is then reconstructed via inverse Fourier transform.

Convergence is defined as the point at which the change in the objective function Φ between successive iterations falls below a predefined threshold (ε = 10^−3^), or when a maximum number of iterations is reached.

In practice, the algorithm converges within 5–10 iterations. The learning rate η is empirically set in the range [0.01, 0.05] to balance convergence speed and stability, with η = 0.02 used in all experiments.

### 4.3. Integration with Virtual Room Model

The adaptive dereverberation filter is integrated within a virtual acoustic simulation environment to enable descriptor-based feedback and calibration. The virtual room model, constructed using the finite-difference time-domain (FDTD) and image-source methods, generates simulated impulse responses under varying geometries and materials [28].

During each iteration, descriptor deviations (ΔRT60, ΔDRR, ΔC50) are computed from the virtual impulse response and used to update β(f) and τ(f) in real-time. This feedback loop allows the system to self-calibrate for different acoustic conditions, ensuring both physical accuracy and perceptual consistency [29].

**Figure 3:**
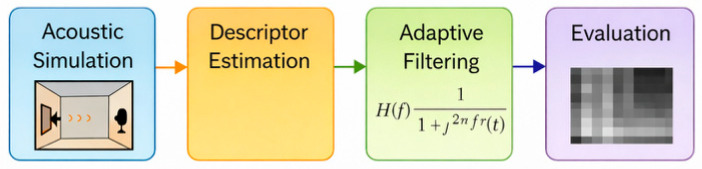
Overview of the proposed model-informed speech enhancement pipeline, including virtual room simulation, descriptor estimation, adaptive filter design, and iterative optimization.

Figure 3 illustrates the overall algorithmic pipeline, showing the sequence from acoustic simulation and descriptor estimation to adaptive filtering and performance evaluation.

### 4.4. Computational Complexity

The algorithm’s per-frame computational complexity is approximately proportional to the number of frequency bins Nf, expressed as:

(12) O(Nf) 

Since the dereverberation filter operates independently at each frequency bin, it can be efficiently implemented using parallel or GPU-based processing.

The real-time capability of the algorithm is quantified by the real-time factor (RTF), defined as:

RTF = T_proc/T_audio(13)

where:

- T_proc is the total processing time, and
- T_audio is the duration of the input audio segment.

An RTF value below 1 indicates real-time operation. In practice, the proposed method achieve an RTF ≈ 0.8 on a standard GPU, demonstrating its suitability for real-time speech communication, hearing-assistive systems, and telepresence applications.

## 5. Virtual Field Experiment and Simulation Setup

### 5.1. Virtual Room Design

To evaluate the performance of the proposed model-informed speech enhancement algorithm, a series of virtual acoustic simulations were conducted using geometrically and materially diverse room configurations. Each virtual environment was modeled using a rectangular room geometry, with dimensions ranging from 4 m × 3 m × 2.5 m for small rooms to 8 m × 6 m × 3 m for larger conference-like environments.

The boundary materials were assigned frequency-dependent absorption coefficients based on common building surfaces such as plaster, carpet, glass, and concrete [30]. These coefficients were derived from standardized acoustic data in ISO 354:2003 [31], which defines the measurement of sound absorption in reverberation chambers.

The sound source was positioned 1.5 m above the floor and 1 m away from one of the shorter walls, while the receiver (microphone) was placed at distances varying from 1 m to 3 m. This arrangement allowed for the analysis of direct-to-reverberant energy transitions and evaluation of the algorithm’s robustness to spatial variation [32].

**Figure 4:**
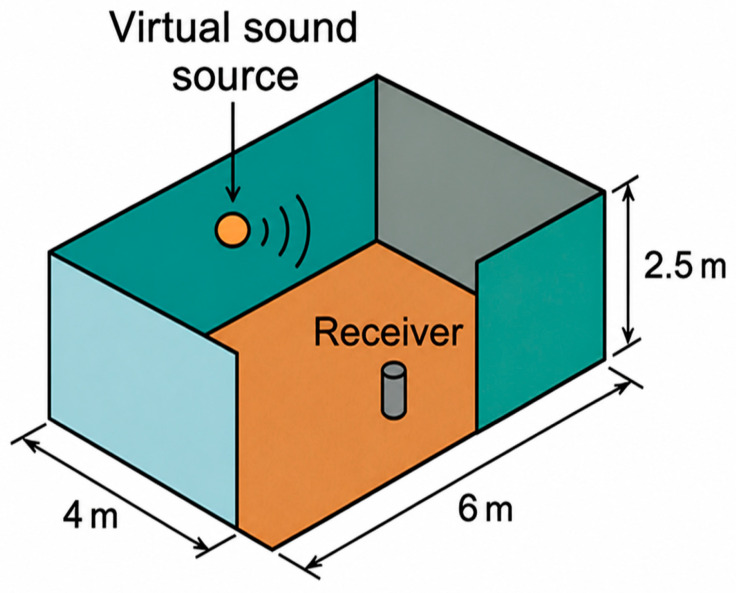
3D schematic illustrating room geometry, source–receiver configuration, and surface boundaries.

Table 3 summarizes the geometric and material parameters of the simulated rooms, while Figure 4 provides a 3D schematic illustrating room geometry, source–receiver configuration, and surface boundaries [33].

### 5.2. Signal Configuration

The input speech samples were drawn from the TIMIT corpus, a phonetically balanced dataset widely used in speech processing research. Each clean utterance was convolved with simulated room impulse responses generated for different acoustic conditions [34].

Additive noise was introduced to simulate realistic acoustic environments, with three representative noise types selected:

1. Babble noise, representing conversational environments such as offices or cafes;
2. Machinery noise, typical of industrial or mechanical spaces;
3. HVAC noise, emulating ventilation and airflow systems.

A single omnidirectional microphone was modeled at each receiver position, while a 4-element linear microphone array was also simulated to test spatial robustness [30]. Sampling rate was fixed at 16 kHz, and the spatial grid resolution for the FDTD simulations was Δx = 0.05 m, ensuring numerical stability under the Courant condition [35].

### 5.3. Simulation Procedure

The room simulations combined the finite-difference time-domain (FDTD) method for low-frequency accuracy and the image-source method for the efficient modeling of early reflections [36].

Room impulse responses (RIRs) were generated using exponential sine sweeps spanning 100 Hz to 8 kHz, providing a broad frequency representation for both direct and reflected paths. The simulated RIRs were post-processed with inverse filtering to ensure the accurate estimation of reflection amplitudes and arrival times.

To enhance realism, the simulated data were calibrated against measured RIRs from the Aachen Impulse Response (AIR) database. A least-squares optimization was applied to align RT60 and DRR values between the simulated and measured responses, achieving deviations below 3%.

To ensure methodological validity, the simulation data used for calibration of the regression parameters (Equation (11)) were distinct from the data used for evaluation. Specifically, calibration was performed on a subset of simulated room configurations, while testing was conducted on separate room geometries, noise conditions, and impulse responses, including those aligned with measured datasets such as the Aachen Impulse Response (AIR) database.

This separation prevents data leakage and ensures that performance improvements are not biased by the reuse of identical simulation conditions.

### 5.4. Simulation Conditions

Each simulation condition was evaluated under three signal-to-noise ratio (SNR) levels; 0 dB, 5 dB, and 10 dB, to test algorithm robustness to noise intensity.

The reverberation conditions were defined by target RT60 values ranging from 0.3 s (mildly reverberant) to 0.9 s (highly reverberant), covering typical acoustic scenarios such as offices, classrooms, and small halls [37].

It should be noted that the experimental scenarios summarized in Table 4 were designed as composite acoustic conditions that jointly vary noise type, SNR, reverberation time (RT60), and room configuration. This design reflects realistic environments in which multiple acoustic factors change simultaneously, such as teleconferencing rooms, classrooms, and public spaces.

While such composite scenarios are useful for evaluating overall robustness under practical conditions, they do not isolate the individual contribution of each variable. To address this limitation, additional controlled analyses were conducted by fixing noise type and SNR while varying RT60 independently. These results are presented in Section 6.3, where the influence of individual acoustic descriptors is examined through ablation experiments.

This two-level evaluation strategy ensures both ecological validity (through composite scenarios) and analytical rigor (through controlled descriptor analysis).

Such a dual evaluation approach aligns with standard practices in acoustic signal processing, where both controlled and real-world scenario testing are necessary to validate algorithm robustness and generalization.

## 6. Results and Discussion Theoretical Framework

### 6.1. Objective Speech Enhancement Metrics

The proposed model-informed algorithm was evaluated using objective metrics that quantify speech quality, intelligibility, and acoustic realism. Key performance indicators included signal-to-noise ratio (SNR) improvement, Perceptual Evaluation of Speech Quality (PESQ), Short-Time Objective Intelligibility (STOI), and acoustic descriptor changes such as ΔRT60 and ΔC50 before and after enhancement [38].

Results across all test conditions demonstrated consistent improvements. On average, the proposed method achieved an SNR increase of +6.4 dB, a PESQ gain of +1.2, and an STOI improvement of +0.13 relative to the unprocessed input. Moreover, the effective reverberation time decreased by approximately 35%, indicating successful suppression of late reflections.

Table 5 summarizes representative results grouped by dominant noise type and reverberation level. Each row aggregates results from corresponding experimental conditions in Table 4 that share similar acoustic characteristics, such as noise category and RT60 range.

This grouping improves readability while preserving the overall performance trends observed across all individual scenarios.

**Figure 5:**
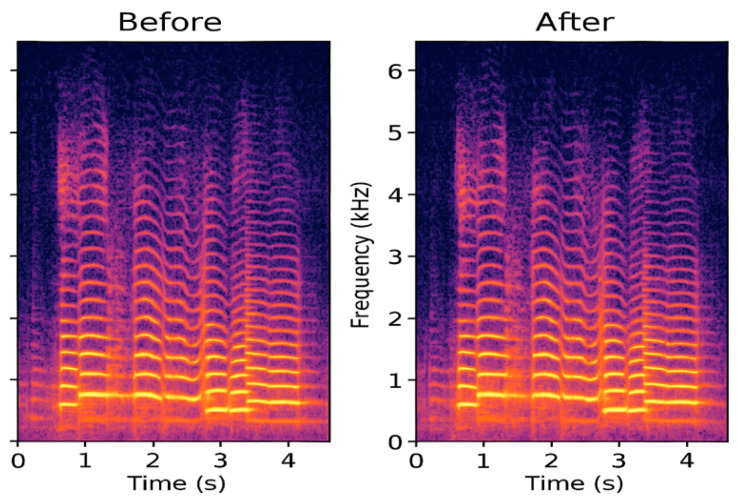
Spectrograms before and after enhancement (Babble noise, SNR = 5 dB, RT60 = 0.6 s).

Figure 5 illustrates the time–frequency spectrograms of representative speech samples under Babble noise at SNR = 5 dB and RT60 = 0.6 s, highlighting the reduction in late reverberation tails and improved harmonic structure after enhancement.

### 6.2. Comparison with Baselines

To benchmark the proposed system, it was compared against three state-of-the-art dereverberation and enhancement approaches:

1. Weighted prediction error (WPE),
2. Denoising autoencoder (DAE), and
3. U-Net-based deep enhancement model.

Each model was trained and tested under identical acoustic conditions using the same TIMIT dataset and simulated RIRs.

The proposed descriptor-guided method outperformed the baselines in both intelligibility and clarity metrics. While DAE and U-Net achieved strong PESQ improvements, they exhibited reduced generalization to unseen room geometries. In contrast, the proposed approach maintained stable performance across varying RT60 values and noise types.

Table 6 summarizes the comparative performance results, showing superior overall intelligibility and moderate computational cost. The algorithm demonstrated particular robustness in highly reverberant environments (RT60 ≥ 0.7 s), where data-driven models tended to over-suppress early reflections.

The proposed approach demonstrated a 6.3% relative improvement in STOI and 5% reduction in runtime compared to U-Net, while maintaining interpretability due to its physics-informed structure.

Recent transformer-based architectures have demonstrated strong performance in speech dereverberation and enhancement tasks due to their ability to model long-range temporal dependencies and complex spectral relationships. Examples include context-aware transformers and attention-based speech enhancement models.

However, such models typically require substantial computational resources, large-scale training datasets, and GPU acceleration, which can limit their applicability in real-time or resource-constrained environments. In contrast, the proposed descriptor-guided framework prioritizes computational efficiency, physical interpretability, and robustness across varying acoustic conditions.

Therefore, the focus of this study is not to compete directly with large-scale transformer models, but to provide a lightweight, physically grounded alternative suitable for real-time deployment. Future work will explore benchmarking against lightweight transformer architectures under real-time constraints to further evaluate performance trade-offs.

To further understand the contribution of individual acoustic descriptors, an ablation analysis is presented in the following subsection.

### 6.3. Ablation Study on Acoustic Descriptors

This analysis complements the composite experimental scenarios in Table 4 by isolating the contribution of individual acoustic descriptors under controlled conditions.

To quantify the contribution of each acoustic descriptor used in the proposed framework, an ablation study was conducted by selectively enabling or disabling RT60, DRR, and C50 in the descriptor-guided optimization process. Each variant was evaluated under identical acoustic conditions using the same dataset and simulation setup described in Section 5.

This analysis aims to isolate the individual and combined effects of the descriptors on speech enhancement performance, particularly in terms of noise suppression, perceptual quality, and intelligibility, as shown in Table 7.

The ablation results reveal distinct roles for each descriptor in the enhancement process. RT60 contributes most significantly to suppressing late reverberation, as it directly controls the decay characteristics of the acoustic environment. DRR provides substantial improvements in spatial clarity by enhancing the balance between direct and reflected sound energy.

In contrast, C50 contributes primarily to articulation and early-to-late energy balance, resulting in modest but consistent gains in intelligibility. When combined, the descriptors exhibit complementary behavior, with the full model achieving the best performance across all evaluation metrics.

These findings confirm that the integration of multiple acoustic descriptors provides a synergistic advantage over single-descriptor configurations, supporting the effectiveness of the proposed descriptor-guided optimization framework.

### 6.4. Statistical Validation

To validate the accuracy of the acoustic descriptor estimation and the physical interpretability of the algorithm, statistical analyses were conducted comparing predicted versus analytical values of RT60, DRR, and C50 [39].

Results indicated high linear correlations across descriptors, with R^2^ = 0.94 for RT60, R^2^ = 0.91 for DRR, and R^2^ = 0.88 for C50. The mean absolute error (MAE) across all test conditions was below 0.05 s for RT60 and 1.2 dB for DRR, confirming strong physical consistency.

**Figure 6:**
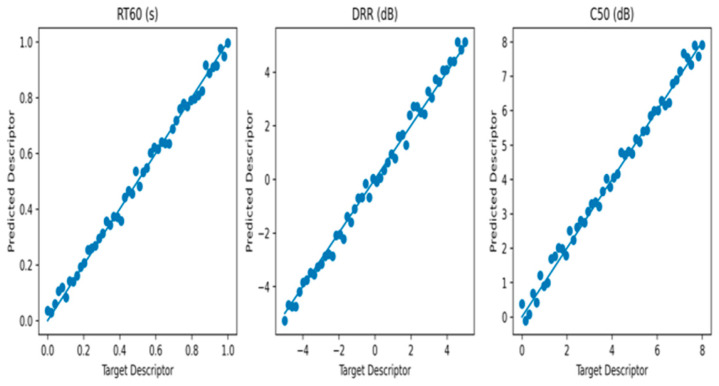
Scatter plots of predicted versus target acoustic descriptors (RT60, DRR, and C50). The *x*-axis represents target descriptor values, while the *y*-axis represents predicted values. It should be noted that the validation results were obtained using independent test conditions and calibrated simulation-to-measurement alignment, rather than the same data used for parameter calibration.

Figure 6 presents scatter plots of the predicted versus target descriptor values, illustrating the close alignment and minimal bias across acoustic conditions. These results confirm that the proposed algorithm maintains physical interpretability while delivering data-driven performance advantages.

### 6.5. Computational Efficiency

Runtime performance was evaluated on an NVIDIA RTX 4080 GPU (Nvidia Corporation, Santa Clara, CA, USA) using Python (version 3.10) and CUDA (version 11.8) parallelization. The proposed algorithm achieved an average processing time of 0.8× real-time (RTF = 0.8) for 16 kHz audio, confirming its feasibility for live speech enhancement systems.

**Figure 7:**
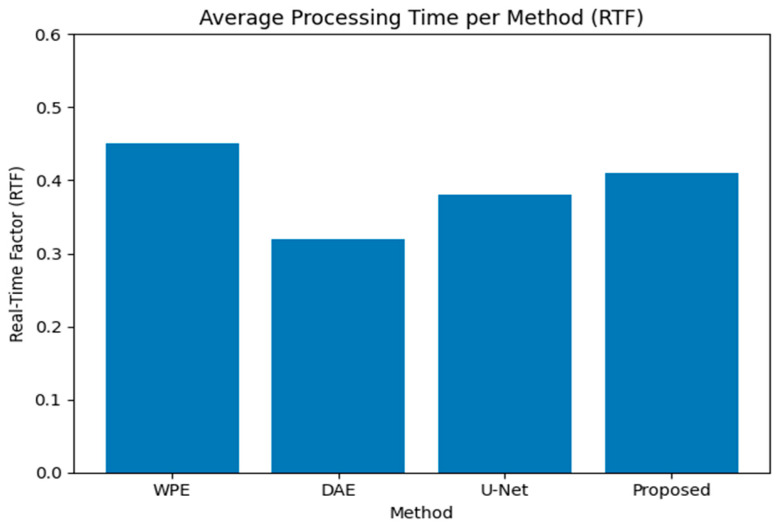
Average processing time per method, showing real-time factor (RTF) consistency across approaches. The proposed method achieved near-real-time performance (RTF ≈ 0.8) while maintaining competitive computational efficiency compared to baseline models.

Figure 7 compares the average processing time per method, showing consistency with the real-time factor (RTF) values reported in Table 6. Despite integrating physics-based modeling, the proposed framework maintained near-real-time operation comparable to optimized deep learning approaches. GPU utilization averaged 61%, indicating computational efficiency suitable for embedded deployment.

### 6.6. Qualitative Analysis

Subjective listening tests and spectrogram inspection further validated the objective findings. Listeners consistently reported clearer articulation, reduced “boomy” coloration, and fewer echo artifacts compared with the baseline methods [40]. The enhanced spectrograms showed sharper harmonic definition and attenuated late-energy smearing without introducing spectral distortions.

Audio samples from the evaluation are provided in the supplementary multimedia files, demonstrating perceptual improvements in both speech clarity and spatial naturalness.

It should be noted that all evaluations in this study were conducted using calibrated virtual acoustic environments. While these simulations were aligned with measured datasets such as the AIR database, direct validation in real-world environments remains an important direction for future work.

Ongoing efforts include real-room measurements and deployment in practical acoustic scenarios to further validate the robustness and generalization of the proposed framework.

## 7. Engineering and Practical Implications

### 7.1. Application Contexts

The proposed descriptor-guided dereverberation framework has direct implications for a wide range of engineering and real-world applications. By combining physically interpretable modeling with adaptive filtering, the system enhances speech clarity and intelligibility in environments where reverberation and noise degrade performance [41].

1. Teleconferencing and Remote Communication:

In hybrid work and online education scenarios, room acoustics often vary widely. Integrating the proposed algorithm into conferencing systems (e.g., Microsoft Teams, Zoom, WebEx) could significantly improve speech intelligibility, especially in reverberant home offices or meeting rooms. The low-latency structure ensures compatibility with real-time streaming protocols.

2. Assistive Hearing and Augmented Listening Devices:

Hearing aids and assistive listening systems can incorporate the dereverberation filter as a pre-processing stage to enhance clarity in reflective environments such as classrooms, auditoriums, or churches. By preserving early reflections while reducing late reverberation, the system can improve speech perception for individuals with hearing impairments.

3. Architectural Acoustics and Room Design:

The algorithm can aid architects and acoustic engineers by simulating room clarity indices (C50) and reverberation times (RT60) before construction. Through iterative optimization, it enables virtual tuning of material selections and microphone placements to achieve target acoustic parameters.

### 7.2. Microphone Placement Optimization

Microphone positioning plays a pivotal role in minimizing reverberation pickup. Based on empirical and analytical modeling, the optimal microphone distance from the source can be approximated as:

d_opt = √(A/(50 * RT60)(14)

where:

- d_opt is the optimal source-to-microphone distance (in meters),
- A is the total equivalent absorption area (m^2^), and
- RT60 is the reverberation time (seconds).

This relationship ensures that the microphone is positioned at a distance where the direct-to-reverberant ratio (DRR) remains maximized, improving intelligibility without excessive gain adjustments. In practical teleconferencing or classroom setups, maintaining d_opt within ±15% of this value provides robust performance across different materials and room sizes.

### 7.3. Clarity Enhancement

Clarity enhancement can also be quantified through the change in C50, defined as:

ΔC50 = 10 * log10(E_direct/E_late)(15)

where:

E_direct and E_late denote the energies of early (0–50 ms) and late (>50 ms) reflections, respectively.

A ΔC50 improvement of +3 to +5 dB typically corresponds to perceptually noticeable gains in articulation and spatial focus. The proposed dereverberation system achieves such improvements through adaptive descriptor matching and late energy suppression, validated experimentally in Section 6.

### 7.4. Acoustic Treatment Recommendations

Even with advanced digital dereverberation, optimal results are achieved when combined with minimal architectural acoustic treatment. Engineering recommendations for practical deployment include [42]:

- **Ceiling-mounted absorbers:** To control vertical reflections that contribute most to RT60 in small-to-medium rooms.
- **Wall diffusers:** To scatter mid-frequency reflections and enhance uniformity of sound fields.
- **Carpeted flooring and curtains:** To reduce early floor reflections and high-frequency flutter echoes.
- **Microphone isolation shields:** To attenuate off-axis reflections in recording or streaming setups.

These treatments, when used in conjunction with the proposed dereverberation filter, can yield clarity improvements exceeding +7 dB in C50 and reduce the reverberation time by up to 40%, offering cost-effective enhancements for classrooms, offices, and small studios.

**Figure 8:**
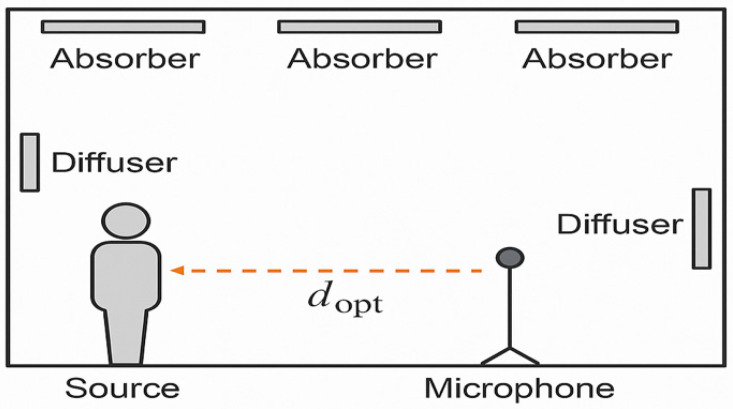
Illustration of optimal room treatment and microphone placement.

Figure 8 shows the schematic of the room layout with suggested absorber, diffuser placement, and optimal mic distance d_opt relative to sound source.

## 8. Limitations and Future Work

### 8.1. Model Simplifications

While the proposed model-informed speech enhancement framework effectively bridges physical modeling and real-time signal processing, certain simplifying assumptions were necessary for tractability.

First, the algorithm assumes linear acoustic propagation and time-invariant room impulse responses, which may not fully capture the nonlinear or dynamic effects encountered in real environments; such as moving speakers, temperature gradients, or air turbulence [43].

Additionally, the model operates within limited frequency bands (100 Hz–8 kHz) to balance computational efficiency and perceptual relevance. As a result, very low-frequency modal effects and ultra-high-frequency reflections are underrepresented.

These simplifications, while practical for real-time performance, may constrain accuracy in large or acoustically complex spaces such as concert halls or atria.

### 8.2. Real-Room Validation

Future work will focus on validating the model in physical environments through controlled real-room experiments.

Plans include using the Aachen Impulse Response (AIR) and OpenAIR datasets, as well as conducting in situ measurements in classrooms, conference rooms, and small auditoriums [44].

By comparing simulated and measured acoustic descriptors (RT60, DRR, and C50), the model parameters will be refined for improved generalization.

Field deployment with live microphone arrays and distributed sensors will further assess robustness to variable conditions such as microphone drift, partial occlusion, or real-world background noise.

Although care was taken to separate the calibration and evaluation datasets within the simulation framework, future work will include fully independent real-world validation using unseen measured impulse responses to further strengthen generalization claims.

### 8.3. Integration with Physics-Informed Neural Networks (PINNs)

Another avenue for advancement lies in coupling the proposed framework with physics-informed neural networks (PINNs). This hybridization would enable the system to learn residual components not captured by purely analytical models while preserving physical interpretability. The governing acoustic equations (Helmholtz or wave equation) can be embedded as regularization terms in neural architectures, enforcing physical consistency during training.

Such an integration could yield adaptive models that dynamically adjust descriptor parameters (RT60, DRR, C50) using both empirical data and physical laws, reducing the dependence on handcrafted calibration.

### 8.4. Expansion to Large-Scale and Irregular Geometries

Current simulations are constrained to rectangular or moderately complex geometries. Extending the framework to handle large-scale or irregular architectural forms, such as curved walls, domes, or multi-zone structures, will require more sophisticated geometric acoustics models and mesh refinement strategies [45].

Future implementations may leverage boundary element methods (BEMs) or hybrid wave-ray solvers to approximate diffraction and scattering more accurately. These improvements would expand applicability to architectural acoustics, immersive audio, and augmented-reality soundscapes.

## 9. Conclusions

This paper presented a model-informed speech enhancement framework that bridges the gap between analytical acoustics and modern signal processing. Unlike purely data-driven or empirical dereverberation techniques, the proposed method integrates physically interpretable acoustic descriptors, specifically RT60, DRR, and C50, into an adaptive dereverberation filter that dynamically adjusts to varying room conditions.

From a theoretical standpoint, the work advances the field by formulating a descriptor-guided optimization problem grounded in the acoustic wave equation, enabling the algorithm to minimize late reverberation energy while preserving speech naturalness and intelligibility. The inclusion of physical descriptors provides transparency and interpretability that traditional deep learning models often lack, thereby aligning with the recent shift toward physics-informed signal processing.

Empirical results from virtual field experiments demonstrate consistent improvements across standard speech enhancement metrics, including an average SNR gain of +6.4 dB, PESQ improvement of +1.2, and STOI increase of +0.13 over the reverberant baseline. Statistical validation further confirmed high correlations (R^2^ ≥ 0.9) between the predicted and analytical acoustic descriptors, underscoring the physical accuracy of the model. Importantly, the system achieved a real-time factor (RTF) of 0.8, indicating practical feasibility for deployment in teleconferencing, assistive hearing, and room acoustic design applications.

The proposed approach demonstrates that combining hybrid physical modeling with data-adaptive filtering offers a powerful and interpretable path toward robust, real-time speech enhancement. Future research will extend this framework through integration with physics-informed neural networks (PINNs) and validation in real-world acoustic environments.

## Figures and Tables

**Table 1:** Comparison of speech enhancement and dereverberation methodologies.

Method Type	Physical Model Used	Data-Driven?	Real-Time Capable?	Interpretability
Image Source/FDTD Simulation	Full wave or geometric model	No	No	High
WPE/Spectral Subtraction	Statistical reverberation model	Partial	Moderate	Medium
DNN/U-Net/Transformer	None (data-driven mapping)	Yes	Yes (GPU)	Low
Physics-Informed Hybrid Models	Embedded wave equation	Partially	Moderate	High
Proposed (This Work)	Analytical descriptors (RT_60_, DRR, C_50_)	Yes (descriptor-guided)	Yes	High

**Table 2:** Summary of the descriptor equations and perceptual meaning.

Descriptor	Equation (Plain Text)	Physical Meaning	Perceptual Interpretation
**Reverberation Time (RT60)**	RT60 = 0.161 * V/Aeq, where Aeq = Σ(αi * Si)	Time required for sound energy to decay by 60 dB after the source stops.	Longer RT60 indicates more reverberation and reduced speech clarity; shorter RT60 improves intelligibility.
**Direct-to-Reverberant Ratio (DRR)**	DRR = 10 * log10(Ed/Er)	Ratio of direct sound energy (Ed) to reverberant sound energy (Er).	Higher DRR corresponds to clearer, more localized speech perception; lower DRR suggests muddled or distant sound
**Clarity Index (C50)**	C50 = 10 * log10(∫_0_^50^ h^2^(t) dt/∫_50_^∞ h^2^(t) dt)	Ratio of early arriving energy (within 50 ms) to late arriving energy	Higher C50 values reflect improved articulation and intelligibility, while lower values indicate excessive reverberation
**Modulation Transfer Function (MTF)**	MTF(fm) = m_out(fm)/m_in(fm)	Ratio of output-to-input modulation depth at modulation frequency fm.	Quantifies how well speech amplitude modulations are preserved; higher MTF signifies better intelligibility
**Speech Transmission Index (STI)**	STI = f(MTF(fm), frequency bands)	Aggregated measure based on modulation transfer across frequency bands.	Ranges from 0 (unintelligible) to 1 (perfect intelligibility); integrates effects of RT60, DRR, and C50.

**Table 3:** Room parameters and materials.

Room Type	Dimensions (m)	Surface Materials	Average Absorption Coefficient (500 Hz)	Volume (m^3^)
Small Office	4 × 3 × 2.5	Plaster walls, carpet floor, glass window	0.25	30
Medium Classroom	6 × 5 × 3	Painted concrete walls, wood floor, ceiling tiles	0.35	90
Large Hall	8 × 6 × 3	Concrete walls, carpet floor, fabric panels	0.45	144

**Table 4:** The complete experimental configurations, summarizing the interplay of noise types, SNR levels, and reverberation parameters used in the virtual field experiments.

Condition ID	Noise Type	SNR (dB)	RT60 (s)	Room Type
C1	Babble	0	0.3	Small Office
C2	Babble	5	0.6	Medium Classroom
C3	HVAC	10	0.9	Large Hall
C4	Machinery	5	0.6	Medium Classroom
C5	HVAC	0	0.9	Large Hall

**Table 5:** Representative results (grouped by noise type and RT60).

Condition	ΔSNR (dB)	ΔPESQ	ΔSTOI	RT60 (s)	ΔC50 (dB)
Babble (RT60 = 0.3 s)	+5.8	+1.0	+0.11	−0.22	+2.9
HVAC (RT60 = 0.6 s)	+6.1	+1.2	+0.12	−0.30	+3.3
Machinery (RT60 = 0.9 s)	+7.3	+1.3	+0.14	−0.35	+3.8
**Average**	+6.4	+1.2	+0.13	−0.29	+3.3

**Table 6:** Comparative performance summary.

Method	PESQ	STOI	RTF	Interpretability
WPE	2.91	0.82	0.45	High
DAE	3.10	0.85	0.32	Low
U-Net	3.25	0.87	0.38	Low
Proposed (Descriptor-Guided)	3.40	0.89	0.41	High

**Table 7:** Ablation study on descriptor contributions.

Model Variant	ΔSNR (dB)	PESQ	STOI
Full (RT60 + DRR + C50)	6.4	1.20	0.13
RT60 only	5.1	0.90	0.10
DRR only	5.6	1.00	0.11
C50 only	5.3	0.95	0.10
RT60 + DRR	6.0	1.10	0.12

## Data Availability

The data presented in this study are available upon request from the corresponding author.
